# Risk factors for incidental parathyroidectomy during thyroidectomy

**DOI:** 10.1590/S1808-86942012000100009

**Published:** 2015-10-20

**Authors:** Niklas Söderberg Campos, Lívia Petrone Cardoso, Ricardo Tirapelli Tanios, Bruna Craveiro de Oliveira, André Vicente Guimarães, Rogério Aparecido Dedivitis, Luiz Francisco Marcopito

**Affiliations:** a Medical Student – Medical School of the Metropolitan University of Santos - UNIMES, Santos/SP, Brazil.; b PhD in Medicine – Graduate Program in Surgery – Medical School of the University of São Paulo- USP. (Professor of Otorhinolaryngology and Head and Neck Surgery – Metropolitan University of Santos, Santos/SP, Brazil); c MD. Senior Associate Professor - Fundação Lusíada UNILUS.; d Senior Associate Professor – Federal University of São Paulo - UNIFESP, São Paulo. (Full Professor of Collective Health at the Metropolitan University of Santos - UNIMES, Santos). Departments of Otorhinolaryngology and Head and Neck Surgery and Scientific Initiation of the Metropolitan University of Santos - UNIMES, Santos/SP, Brazil.

**Keywords:** parathyroid glands, thyroid neoplasms, thyroidectomy

## Abstract

Incidental parathyroidectomy is a common event in thyroid surgery. The literature shows a finding of parathyroid glands ranging from 6.4% to 31% in pathological specimens of the thyroid gland.

**Objective:**

To collect the amount of parathyroid glands found in surgical specimens of thyroidectomy and correlate with the histopathological and demographic variables.

**Methods:**

Retrospective study based on pathological reports of thyroidectomy from January 2007 to December 2008.

**Results:**

442 patients were submitted to total thyroidectomy, and 2.93% had parathyroid glands, which corresponded to 13 of this total. The presence of papillary thyroid carcinoma associated with incidental parathyroidectomy was 10.11%, compared to the benign lesion: 1.4%.

**Conclusion:**

Papillary thyroid carcinoma was the variable associated with increased number of incidental parathyroidectomy.

## INTRODUCTION

Accidental parathyroidectomy is a frequent occurrence during thyroidectomies. Some studies have assessed the incidence rates of accidental parathyroidectomies, which vary between 6.4% and 31.0%^1^, ^2^, ^3^, ^4^, ^5^, ^6^, ^7^. Most of the times, there are four parathyroid glands. Nonetheless, this number may vary, fluctuating between two and 19^2^. The most commonly location is subcapsulary^1^, ^2^, ^3^, ^4^, ^5^, ^6^, ^7^. These glands develop from the third and fourth pairs of embryonic pharyngeal pockets, and its histology is made up mainly of oxyphil and parathyroid cells^3^. The parathyroid gland vascularization stems from branches of the upper and lower thyroidea arteries^4^, ^5^. During surgery, it is essential to handle it with care, so as to preserve the vascularization of the parathyroid glands^3^.

The rate of complications, such as hematomas, infection, keloid and damage to the recurrent laryngeal nerve during thyroidectomies is 5%^8^, ^9^. The two most common complications are described in decreasing order, such as recurrent laryngeal nerve damage and hypocalcemia^1^, ^2^, ^3^, ^4^, ^5^, ^6^, ^7^.

The goal of the present paper is to identify the risk variables concerning the accidental removal of the parathyroid glands during thyroidectomy.

## METHODS

The present study was approved by the Ethics in Research Committee of the Institution where it was designed, under protocol # 013/2010.

**Figure 1:**
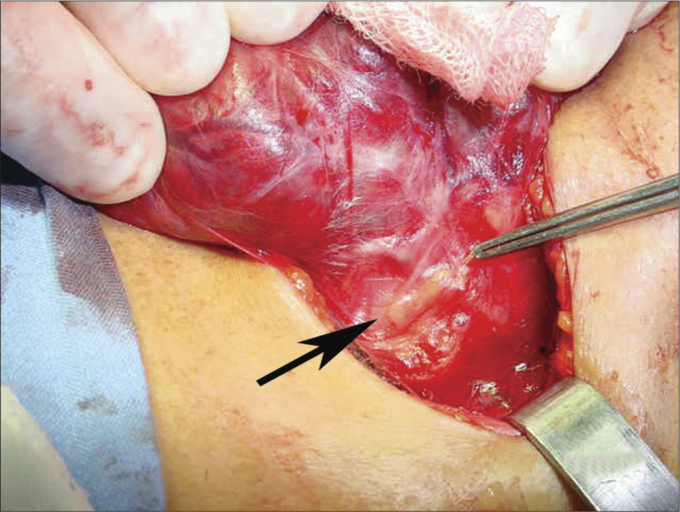
Extracapsular identification of the thyroid gland in the operating field.

We carried out a retrospective study, in which we used 442 charts between January of 2007 and December of 2008. We assessed the demographic variables: age and gender; and the histopathological ones (thyroiditis, histopathology diagnosis, specimen weight, size in its longest axis and the presence of concurrent neck dissection). During the surgical procedure, we systematically tried to identify the parathyroid glands in the surgical field (Figure 1) and in the resected specimen.

According to Table 1, we had a predominance of women (5.5 times more) among patients submitted to thyroidectomy. In relation to the type of procedure to which the patients were submitted to, partial thyroidectomy came in first place – with the largest number of cases, followed by total and, afterwards, totalization. Approximately 10% had thyroiditis. The final diagnosis of the specimen submitted to histopathology varied from colloid goiter – found in greater number, all the way to thyroiditis. Papilliferous carcinoma was present in approximately 20% of the cases. We did recurrent neck dissection in about 2% of the thyroidectomized patients.

**Table 1:** Sample characterization (n = 442).

Variable	Distribution
Age	Mean = 49.6 years
Gender	
Men	68 (15.38)
Women	374 (84.61)

Thyroidectomy	
Partial	234 (52.94)
Total	197 (44.57)
Totalization	14 (3.15)

Thyroidectomy	
Present	41 (9.26)
Absent	404 (91.40)

Diagnosis	
Colloid goiter	82 (18.55)
Adenomatous goiter	73 (16.51)
Nodular goiter	59 (13.34)
Follicular adenoma	52 (11.76)
Thyroiditis	18 (4.07)
Papilliferous carcinoma	89 (20.13)

Neck dissection	8 (1.81)

## RESULTS

The total number of thyroidectomized patients was 442, and parathyroid glands were found in 13 of these, corresponding to 2.93% of all thyroidectomized patients who took part in the study. When broken down by gender, there were 68 males, with four cases of parathyroid glands found in the surgical specimens and, when we calculated the percentage, it resulted in 0.9% - results which were lower than those in the second group – with 9 cases from a total of 374 patients – 2.03%. The general incidence of accidental parathyroidectomies in elective thyroidectomies was 3.5% (13 in 373). Nonetheless, it reached 10.1% (nine in 89) when the indication for thyroidectomy corresponded to papilliferous carcinoma, and when compared to the other indications, which added to 1.4% (four in 284), its association was much more probable. Thus, variables such as: gender, age, having thyroiditis or not and the type of procedure carried out did not influence the finding of accidental PG in surgical specimens. For statistical purposes, we used the bicaudal Fisher's test in order to calculate the likelihood of an association between accidental parathyroidectomies and the TG papilliferous carcinoma being true. It yielded a very low probability (*p* = 0.0005) of such difference in percentage (10.1 *versus* 1.4) having occurred by chance.

As far as the topographic distribution of the parathyroid glands found in surgical specimens is concerned, results showed seven (2.93%) cases in the gland's extracapsular region, five (1.13%) in the subcapsular region and one (0.22) in the intraglan-dular region.

The presence of thyroid papilliferous carcinoma associated with accidental parathyroidectomy was 10.11%; compared to 1.4% of benign disease - Tables 2 and 3.

**Table 2:** Accidental parathyroidectomy in thyroidectomy surgeries in relation to the thyroid gland papilliferous carcinoma.

Accidental parathyroidectomy			
Indication of thyroidectomy	Yes	No	Total (n)
Papilliferous carcinoma	9	80	89
Others	4	280	284
Total (n)	13	360	373

**Table 3:** Description of the cases of accidental parathyroidectomy.

Patient	Age (years)	Gender	Diagnosis	Type of thyroidectomy	thyroiditis
1	48	Male	Papilliferous carcinoma	Partial	No
2	68	Male	Papilliferous carcinoma	Partial	No
3	54	Male	Papilliferous carcinoma	Partial	No
4	36	Male	Papilliferous carcinoma	Partial	No
5	33	Female	colloid goiter	total	No
6	39	Female	Papilliferous carcinoma	total	No
7	41	Female	colloid goiter	Partial	No
8	48	Female	colloid goiter	Partial	No
9	57	Female	Thyroiditis	Partial	Yes
10	25	Female	Papilliferous carcinoma	total	No
11	26	Female	Papilliferous carcinoma	Partial	No
12	68	Female	Papilliferous carcinoma	total	No
13	40	Female	Papilliferous carcinoma	Partial	No

## DISCUSSION

Thyroidectomy is a relatively safe surgical procedure, and its main complications include injury to the parathyroids, potentially manifested by temporary or permanent hypocalcemia^4^.

Accidental parathyroidectomy is a frequent finding, even in experienced hands. Since the XIX century, thyroid surgeries have had a reduction in the incidence of complications^9^. Following the literature, the incidence of accidental parathyroidectomy varies between 5.2% and 31% during thyroidectomies^1^, ^2^, ^3^, ^4^, ^5^, ^6^, ^7^, ^8^, ^9^, ^10^ - Table 4. Nonetheless, surgeon experience in thyroid surgery has been a determining factor in the better identification and preservation of parathyroid glands during thyroidectomy^8^, minimizing such complication. Although in our clinic the surgical team is made up of residents and surgeons with 4 to 12 years of practice, our results concerning accidental parathyroidectomy was of only 2.93%.

**Table 4:** Accidental parathyroidectomy incidence in the literature.

Authors, year	Number of patients	n / %
Erbil et al., 20096	440	48 / 10,9
Manouras et al., 20083	508	100 / 19,7
Sippel et al., 20075	513	33 / 6,4
Page et al., 200710	351	18 / 5,2
Gourgiotis et al., 20061	315	68 / 21,6
Sakorafas et al., 20054	158	28 / 17,7
Sasson et al., 20017	141	21 / 15

Familiarity with the parathyroid anatomy and its blood supply system is necessary to prevent inadvertently injuring it, devascularization or resection of parathyroid parenchyma^11^. The variability in its location may contribute to the risk of accidental avulsion.

Most parathyroid glands have extracapsular location^1^, ^2^, ^3^, ^4^, ^5^, ^6^, ^7^, ^8^, ^9^, ^10^, which enables its identification and preservation. 49% of the glands have been found inside the thyroid and, in such cases, it is impossible to spare it^3^.

Some authors advocate the systematic identification of the glands in the surgical field^12^, while others believe that the juxta-capsular dissection of the thyroid with the ligature of small vessels to be the best way to see the parathyroids and their blood supply^10^. Special care must be taken near the upper parathyroid, which is frequently located near the recurrent laryngeal nerve as it enters the lower thyroidea artery^10^.

The most relevant risk factor was the presence of thyroid papilliferous carcinoma (10.11%) against the presence of benign disease (1.4%). One possible explanation for this finding would be the need to increase the oncologic surgical margin and, sometimes, including a probable metastatic lymph node in the surgical specimen. Theoretically, the risk of accidental parathyroidectomy may increase in some situations, such as in patients submitted to extensive surgery by malignant disease, in the presence of extra-thyroid extension, or in the presence of a large lymph node metastasis in the surgical specimen. In this direction, modified radical neck dissection has been recognized as a risk factor^7^. Special attention when doing the dissection in the neck central compartment may reduce the risk of such complication; nonetheless, oncological procedures must not be compromised.

Thyroidectomy totalization and reoperations have been correlated with the increase in the rates of accidental parathyroidectomies, probably as a result of fibrosis, which may cause operatory difficulty. Thyroiditis may also have been described as another risk factor^13^.

A careful inspection of the surgical specimen in search of the normal parathyroid gland tissue is prudent, and it may lead to auto-transplant. Such practice must be routine, especially when more than two glands are identified in these conditions, or when the preservation of vascularization of the other glands is questionable. Routine auto-transplant results in less than 1% incidence of permanent hypoparathyroidism^14^, ^15^. In fact, the impact on the serum levels of calcium will be lower when only one gland is resected, and the others are preserved^10^. The surgical field must also be carefully assessed, with the aim of certifying the feasibility of the preserved glands. Auto-transplant is also indicated in cases of persistent isquemia^10^.

## CONCLUSION

Papilliferous carcinoma was the variable associated with accidental parathyroidectomy.
